# Long-term decline of marine viruses associated with warming and oligotrophication at a NW Mediterranean coastal site

**DOI:** 10.1093/ismeco/ycaf150

**Published:** 2025-08-29

**Authors:** Xabier Lopez-Alforja, Elisabet L Sà, Maria V Quiroga, Massimo C Pernice, Clara Cardelús, Vanessa Balagué, Josep M Gasol, Felipe H Coutinho, Ramon Massana, Dolors Vaqué

**Affiliations:** Department of Marine Biology and Oceanography, Institut de Ciències del Mar (ICM-CSIC), 08003 Barcelona, Catalonia, Spain; Department of Genetics and Microbiology, Autonomous University of Barcelona, 08193 Bellaterra, Catalonia, Spain; Department of Marine Biology and Oceanography, Institut de Ciències del Mar (ICM-CSIC), 08003 Barcelona, Catalonia, Spain; Instituto Tecnológico de Chascomús (CONICET-UNSAM), Escuela de Bio y Nanotecnologias (UNSAM), 7130 Chascomús, Buenos Aires, Argentina; Department of Marine Biology and Oceanography, Institut de Ciències del Mar (ICM-CSIC), 08003 Barcelona, Catalonia, Spain; Department of Marine Biology and Oceanography, Institut de Ciències del Mar (ICM-CSIC), 08003 Barcelona, Catalonia, Spain; Department of Marine Biology and Oceanography, Institut de Ciències del Mar (ICM-CSIC), 08003 Barcelona, Catalonia, Spain; Department of Marine Biology and Oceanography, Institut de Ciències del Mar (ICM-CSIC), 08003 Barcelona, Catalonia, Spain; Department of Marine Biology and Oceanography, Institut de Ciències del Mar (ICM-CSIC), 08003 Barcelona, Catalonia, Spain; Department of Marine Biology and Oceanography, Institut de Ciències del Mar (ICM-CSIC), 08003 Barcelona, Catalonia, Spain; Department of Marine Biology and Oceanography, Institut de Ciències del Mar (ICM-CSIC), 08003 Barcelona, Catalonia, Spain

**Keywords:** marine viruses, marine microbial ecology, time series, climate change, machine learning, generalized additive mixed models (GAMM), coastal waters, NW Mediterranean

## Abstract

Viruses play key roles in controlling microbial abundance and community composition, nutrient cycling, and productivity in marine systems. Rising ocean temperatures, alongside increasing oligotrophy, are expected to alter the availability of inorganic nutrients and oxygen—key environmental factors that shape microbial community structure and virus-host interactions. While many studies have investigated viral abundances and community structure across spatial gradients, less is known about their long-term temporal variations, which is particularly relevant in the current context of global change. To address this gap, we analyzed two decades of surface water data from the Blanes Bay Microbial Observatory, located at the North-Western Mediterranean, to describe how biotic and abiotic variables influence temporal dynamics of viral abundances and community composition. Statistical tools for time series, including GAMMs, anomaly analysis, and neural networks, allowed us to demonstrate that viral abundance follows strong seasonality and a clear decrease starting midway (ca. 2011) through the sampled period (2005–2022). Fingerprint analysis evidenced that viral community composition was significantly influenced by seasonality and some environmental and biotic factors, with strong differences in viral communities between summer and winter months. Our analyses revealed that over the last 18 years, the abundance of most microbial groups, including viruses and their potential hosts, has declined, coinciding with an increase in seawater temperature and transparency, as well as a notable decrease in nutrient concentrations and phytoplankton biomass. We identified the ongoing shift toward more oligotrophic conditions as a potential driver of the observed decline in viral abundance, particularly in the last decade.

## Introduction

Coastal ecosystems harbor diverse microbial communities, including viruses, heterotrophic, and phototrophic protists, as well as prokaryotes (bacteria and archaea) [1]. Viruses are highly abundant and play a fundamental role in regulating the abundance and diversity of marine microorganisms. Viral infections often culminate in host cell lysis, releasing dissolved and particulate organic matter that fuels the microbial food web [2]. This process, known as the viral shunt, redirects energy and nutrients within the microbial loop. Beyond this recycling role, viruses also contribute to the viral shuttle, enhancing the export of organic matter to deeper ocean layers, and thereby influencing long-term carbon sequestration [3].

Global climate change has led to a notable increase in seawater temperatures, as accurately described in the most recent Intergovernmental Panel on Climate Change report (AR6; [4]). In fact, the western Mediterranean Sea has been identified as a hotspot of temperature increase [5], experiencing not only record-breaking temperatures due to more intense warming, but also an increase in the frequency and severity of heatwaves [6, 7]. These changes have notable impacts on marine ecosystems, affecting the physiology, distribution, and interactions of microbial populations [8]. Then, since viruses play a pivotal role in microbial dynamics, understanding how global climate change-driven processes alter marine viral communities is crucial.

Although numerous studies have explored the spatial variations in the abundances and community composition of marine viruses, less effort has been devoted to describing temporal variations. Indeed, the few existing time-series analyses have been short, typically less than 2 years in duration [9–11]. As of the date of this research, there is no known work on time series of viral abundance covering periods longer than a decade, and even less for viral community composition. For instance, Wang et al. (2011) described the abundance and distribution of *Synechococcus* and cyanophages in the Chesapeake Bay over a 5-year period [12], while Li & Dickie (2001) [13] monitored virioplankton abundance weekly for 8 years in the Bedford Basin. Similarly, Parsons et al. (2012) studied recurrent seasonal patterns of virioplankton at a Sargasso Sea station over a 10-year period [14]. These relatively long time series have mainly focused in the northwestern Atlantic, while in the Mediterranean Sea the longest study covers 2 years [15]. This is particularly relevant, as different Longhurst regions might have specific oceanographic conditions and could be much more susceptible to long-term changes due to climate change. Hence, there is a notable gap in long-term Mediterranean virioplankton studies aiming to model viral abundance and understand its temporal variability over extended periods.

Most of the current studies on the relationship between viral abundance and environmental parameters typically use correlations or linear regressions [9, 14, 16], which may be inadequate to capture the non-linear, time-dependent dynamics of viral/microbial communities [17]. Time-varying environmental factors could potentially influence the relative abundance of microbes and viruses, a complexity that has proven challenging for classic time series models. In this context, Generalized Additive Mixed Models (GAMMs) and Artificial Neural Networks (ANNs) emerge as promising and complementary tools. On one hand, GAMMs have been shown to be a versatile non-parametric tool to model time-dependent nonlinearity in microbial systems, addressing some of the current models limitations [18]. On the other hand, ANNs are useful for modeling nonlinear associations using multiple interacting parameters that regulate microbial communities [19–21]. The combined use of GAMMs and ANNs can provide a more comprehensive understanding of the nuanced dynamics of marine viral abundance and reveal hidden patterns that may yield valuable insights in the context of climate change.

This study examines nearly 18 years of monthly surface samples from the Blanes Marine Microbial Observatory to investigate the temporal dynamics of viral abundance and 11 years of data on viral community composition. We aim to determine their relationship with seasonal and annual variations, as well as their connections with both abiotic and biotic factors. Our goal is to construct comprehensive models that link viral dynamics with environmental conditions, capturing both short-term fluctuations and long-term trends in the context of global change.

## Materials and methods

### Sampling site and design

Surface seawater samples (0.5 m depth) were collected monthly from the Blanes Bay Microbial Observatory (BBMO, 41° 39.9′ N, 2° 48.3′ E, NW Mediterranean Sea), ~800 m out from the Blanes harbor, from May 2005 to December 2022. The BBMO is located in an open, shallow bay (20 m depth), with relatively low anthropogenic or terrestrial influence. Samples were first filtered through a 200 μm mesh net and then collected in 20 l polycarbonate carboys, which had been acid-cleaned (1% diluted HCl). Subsequently, the carboys were transported to the Institut de Ciències del Mar laboratories as quickly as possible and in the dark to minimize possible alterations induced by light.

### Physicochemical parameters

Temperature and salinity of the seawater were measured directly at the sampling site using a CTD instrument (Conductivity, Temperature, Depth, model SAIV A/S SD204). Transparency of the water column was estimated using a Secchi disk.

#### Inorganic nutrients

Dissolved inorganic nutrients (including NO_3_^−^, NO_2_^−^, and PO_4_^3−^) were quantified in duplicate 10 ml samples using an Alliance Evolution II autoanalyzer, following Hansen & Koroleff (1999) [22] with minor adjustments.

### Biological variables

#### Chlorophyll *a concentration*

Chlorophyll *a* (Chl a) concentration was determined fluorometrically [23] following the acetone extraction protocol (90% acetone, cooled to 4°C 24 h) of the GF/F filters (Whatman) obtained after filtering 250–500 ml of seawater. Fluorescence was subsequently measured using a Turner Designs fluorometer.

#### Microbial abundances by flow cytometry

Two milliliters aliquots were fixed with glutaraldehyde (0.5% final concentration) for viral counts, or with paraformaldehyde (1%) +glutaraldehyde (0.05%) for picocyanobacterial counts [24]. Fixed samples were kept at 4°C for 15 min in the dark, snap frozen in liquid nitrogen, and stored at −80°C [25]. Counts were performed in a FACSCalibur flow cytometer (Becton and Dickinson, Franklin Lakes NJ, USA) for samples to September 2020, and after that with a CytoFLEX SX flow cytometer (Beckman and Coulter, Brea CA, USA), both using a blue laser emitting at 488 nm. For viral counts, aliquots were stained with SYBRGreen I (Molecular Probes, Invitrogen) at a final 5 × 10^−5^ dilution from the commercial stock with TE (10:1 mM Tris:EDTA), and run at medium flow speed to reach a rate between 100 and 800 events s^−1^ [26]. Green fluorescence and side scatter (SSC) data were used to determine Viral abundance using the CellQuest software (Becton and Dickinson, Franklin Lakes NJ, USA) [15]. For picocyanobacterial counts, including *Synechoccocus* and *Prochlorococcus*, unstained samples were run and events recorded based on SSC and pigment properties.

#### Microbial abundances by epifluorescence microscopy

Samples were preserved with glutaraldehyde (1% final concentration). For heterotrophic prokaryotic abundance, subsamples of 5 ml were filtered through 0.2 μm pore-size black polycarbonate filters, while for nanoflagellate counts subsamples of 20 ml were filtered in filters of 0.6 pore-size. Cells were then stained with DAPI (4′,6-diamidino-2-phenylindole, dihydrochloride [27]) at a final concentration of 5 μg ml^−1^ [28]. Abundances were determined using an OlympusBX40-102/E epifluorescence microscope (Olympus Optical Co., Hamburg, Germany) at 1000× magnification and using the UV filter for DAPI stained cells. Nanoflagellates were also inspected by blue light and cells exhibiting red-orange fluorescence were categorized as phototrophic nanoflagellates (PNFs) while those lacking autofluorescence as heterotrophic nanoflagellates (HNFs). We use the term “heterotrophic prokaryotes” for consistency, although DAPI staining with epifluorescence microscopy may also detect non-pigmented autotrophic prokaryotes such as ammonia-oxidizing archaea.

### Viral community composition: random amplified polymorphic DNA

Samples for random amplified polymorphic DNA (RAPD) were collected monthly from September 2011 to December 2021 to obtain a fingerprint of dsDNA viral diversity. For each sample, 5 l of seawater were filtered using 3.0 and 0.22 μm pore-size filters, followed by tangential flow filtration (30 KDa VIVAFLOW 200) to concentrate free viruses to a final volume of 50 ml. The viral concentrate was filtered through a 0.2 μm pore-size polycarbonate filter to eliminate any remaining prokaryotes, and then concentrated to 400 μl by centrifuging at 4500 rpm for 10 min in 30-kDa ultracentrifugal filter tubes (Amicon, Sigma). The detailed protocol, following Winget & Wommack (2008) [29], is explained in the Supplementary Material.

### Statistical analyses

Time series anomaly analysis was performed using the ICES Working Group on Phytoplankton and Microbial Ecology method [30], taking into account Mackas et al.’s (2001) [31] correction to empty data points in the time series. Annual average anomalies were calculated from monthly deviations from the interannual average. Anomalies for a given month *p*′(t) were determined by subtracting the value of each month from its interannual average $\overline{P}$ Equation (1), then averaging these differences to obtain the final annual anomaly (*p*′_annual_; Equation (2)).


(1) \begin{equation*} {p}^{\prime }(t)={\log}_{10}\left[P(t)\right]-{\log}_{10}\left[\overline{P}\right]={\log}_{10}\left[\frac{P(t)}{\overline{P}}\right] \end{equation*}

(2) \begin{equation*} {p}_{\mathrm{annual}}^{\prime }=\frac{1}{12}{\sum}_{t=12}^{12}{p}^{\prime }(t) \end{equation*}

The comparison of RAPD band fingerprints was performed using the *vegan* version 2.6-8 package [32], which facilitated the calculation of Jaccard dissimilarities and the execution of *PERmutational Multivariate ANalysis Of VAriance* (PERMANOVA). The same package was used for *Non-metric Multidimensional Scaling* (NMDS), which enabled the visualization of dissimilarities between viral communities in different seasons. Pairwise comparisons after PERMANOVA were done with *pairwiseAdonis* version 0.4.1 package [33], applying Bonferroni adjustments to ensure robust statistical inference across seasons.

The used package for constructing GAMMs was *mgcv* version 1.9 [34], which provided essential tools for fitting non-linear statistical models. This was particularly useful for exploring non-parametric relationships and modeling complex patterns in the data. For temporal models, the seasonal component of GAMMs captures annual cyclic patterns, while the interannual component reveals long-term trends. Data autocorrelation was accounted for by an aleatory term (autocorrelation structure of order 1 with a continuous time covariate, *corCAR1*). If month-trend interaction was statistically significant, it was included in the model using a tensor product interaction. Quasipoisson and Gaussian families were implemented for modeling biotic and abiotic data, respectively.

For the ANNs, we used *nnet* version 7.3 [35] and *caret* version 6.0 [36]. *nnet* was employed to construct and train the neural networks. Simultaneously, *caret* was used as a comprehensive tool for model training and evaluation, simplifying the comparison of multiple models and facilitating hyperparameter tuning. Model training, hyperparameter tuning, and data analysis were described in the Supplementary Material.

We conducted all analyses using R version 4.3.2 (R Core Team, 2023). For data processing and visualization, we relied on the *tidyverse* version 1.3 packages [37] and *ggplot2* version 3.4.4 [38]. The dplyr version 1.1.4 [39] was used for efficient data management of large datasets.

## Results

### Seasonal patterns and long-term trends in viral abundance

**Figure 1:**
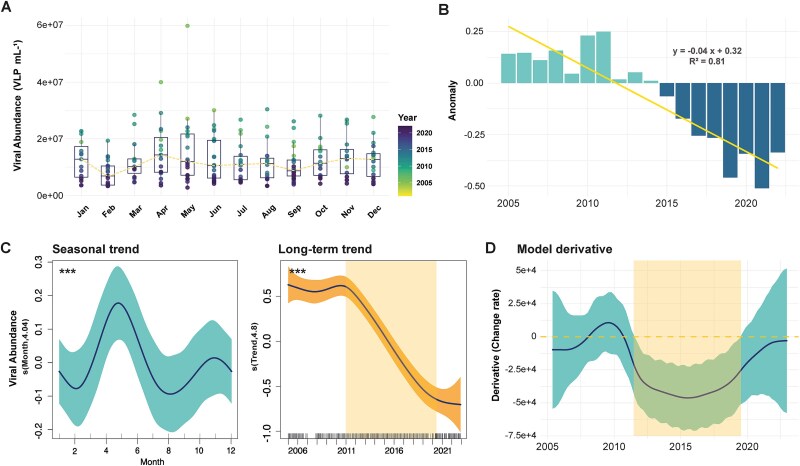
Trends and yearly anomalies in viral abundances. (A) Monthly boxplots of observed viral abundance. The central line represents the median, the box spans the interquartile range (IQR), and the whiskers extend to 1.5 times the IQR. (B) Calculated anomalies of viral abundance, with a linear regression (*N* = 12, *R*^2^ = 0.81) superimposed to show the overall anomaly trend. (C) Partial effect of time on viral abundance modeled by GAMMs, showing the seasonal and long-term trends across the study period. (D) First derivative of the GAMM, representing the rate of change in viral abundance over time, with periods of significant change marked.

Viral abundances in BBMO planktonic surface samples were measured monthly from 2005 to 2022—220 samples. Viral counts ranged from 2.77 × 10^6^ to 5.99 × 10^7^ VLP ml^−1^, with a median value of 1.06 × 10^7^ VLP ml^−1^ (Supplementary Table S1). A clear seasonality was observed, with viral abundance peaking in May (1.58 × 10^7^ ± 0.23 VLP ml^−1^; Fig. 1A and C). During the summer months, viral abundances tended to decrease, followed by a slight increase in November (1.36 × 10^7^ ± 0.14 VLP ml^−1^) before reaching their minimum levels in February (7.72 × 10^6^ ± 0.79 VLP ml^−1^). Long-term trends indicate stable viral abundances from 2005 to 2011 (Fig. 1C), followed by a sharp decline starting in late 2011 until the end of the time series. Indeed, the minimum values ever registered occurred during the last years, with the lowest viral seasonal peak value registered in May 2021 (2.77 × 10^6^ VLP ml^−1^). Anomaly analyses also pinpointed an inflection around 2011–2012, where the trend line crossed the x-axis (*R*^2^ = 0.83; Fig. 1B). GAMM derivatives confirmed a significant negative change rate between 2012 and 2019 (Fig. 1D). A conditional GAMM analysis further identified a significant inflection around 2011–2012, coinciding with a weakening of the seasonality observed in earlier years (Supplementary Fig. S1).

### Temporal changes **in** viral community composition

Viral diversity was assessed over 11 years (2011–2021) on a monthly basis, with a total of 120 samples, using RAPD banding profiles as a proxy for viral community composition. The banding pattern of each sample reflects the viral community composition at the time of sampling. A comparison of bands among samples provided a rough estimation of seasonal and annual changes in viral community composition. We observed four to 17 distinct bands per sample, ranging in size from 100 to 2000 base pairs (Supplementary Fig. S2).

**Figure 2:**
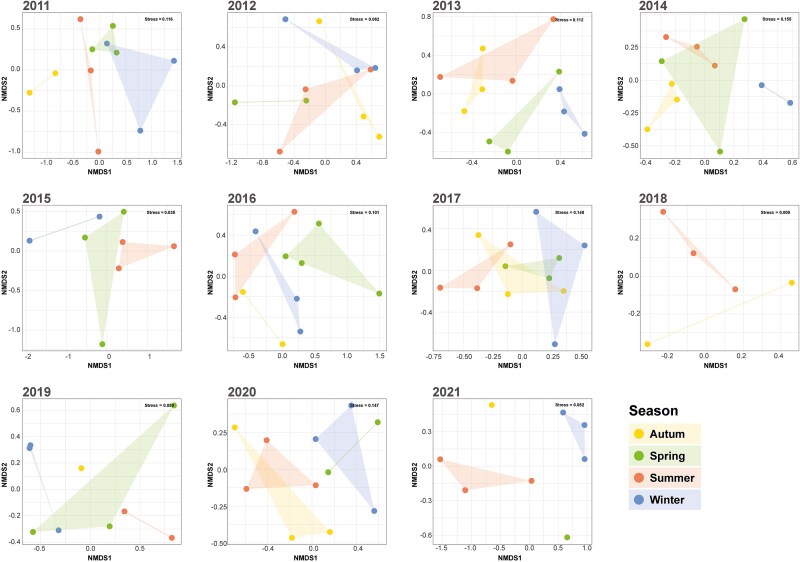
NMDS yearly plots based on Jaccard dissimilarities of band patterns from RAPD analysis of 120 samples collected between 2011 and 2021. Each NMDS represent a different year. The data points are color-coded by season, and polygons are used to outline each season for improved clarity and visualization. Note that summer and winter polygons (almost) never intersect.

Jaccard dissimilarities were calculated among samples based on their RAPD band profiles. The obtained dissimilarity matrix was used as input for NMDS, which displayed a clear separation between winter and summer samples, except for 2016, where no distinct separation was observed, and 2018, where winter data was absent (Fig. 2). This finding was corroborated by PERMANOVA, which revealed a significant effect (*P* < .05) of seasonality nested within years. Furthermore, comparisons of samples grouped by season showed significant differences (*P* < .05) only between summer and winter. However, when analyzing non-nested effects, neither season nor year was found to be significant, even though samples displayed higher richness in the period between 2017 and 2019 (Supplementary Fig. S3).

The associations among environmental and biological variables and viral community composition (RAPD band profiles), were also evaluated through PERMANOVA. Temperature (*P* = .015), and the abundance of PNF (*P* = .032) were the only variables that displayed statistically significant associations with viral community composition. However, a large portion of the variability in the samples could not be explained by these variables (residual variance, *R*^2^ = 0.86).

### A changing coastal system driven by global change

**Figure 3:**
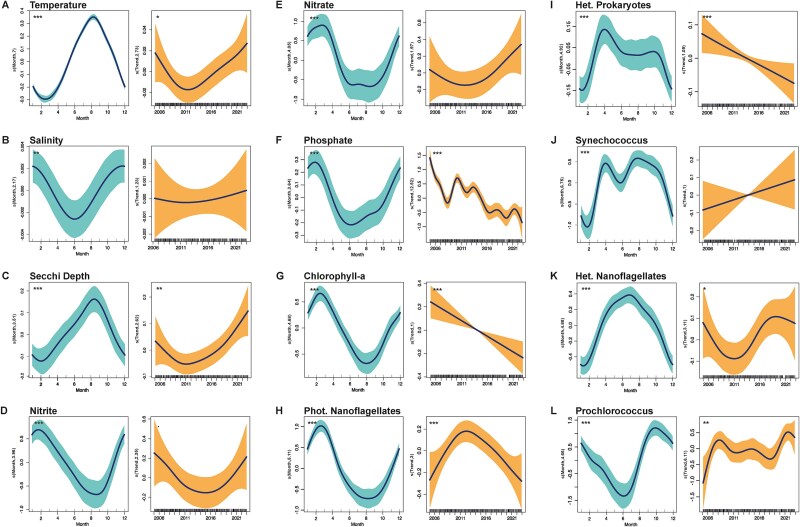
GAMMs for different environmental and biotic variables. Partial effects are modeled using GAMMs considering seasonality (left plots) and long-term variation (right plots) and using as predictor variables: (A) Temperature, (B) Salinity, (C) Secchi depth, (D) Nitrite, (E) Nitrate, (F) Phosphate, (G) Chl a concentration, (H) PNF, (I) Heterotrophic prokaryotes, (J) *Synechococcus*, (K) HNFs, and (L) *Prochlorococcus* abundances. Smoothed terms are centered at zero, facilitating the comparison of the trends. The shaded areas represent the 95% confidence intervals of the smoothed spline functions, highlighting both seasonal variation and long-term trends observed. The statistical relevance of each smoothing term is marked with *P*-value indicators (^***^P < .001, ^**^P < .01, ^*^P < .05, *P* < .1).

**Figure 4:**
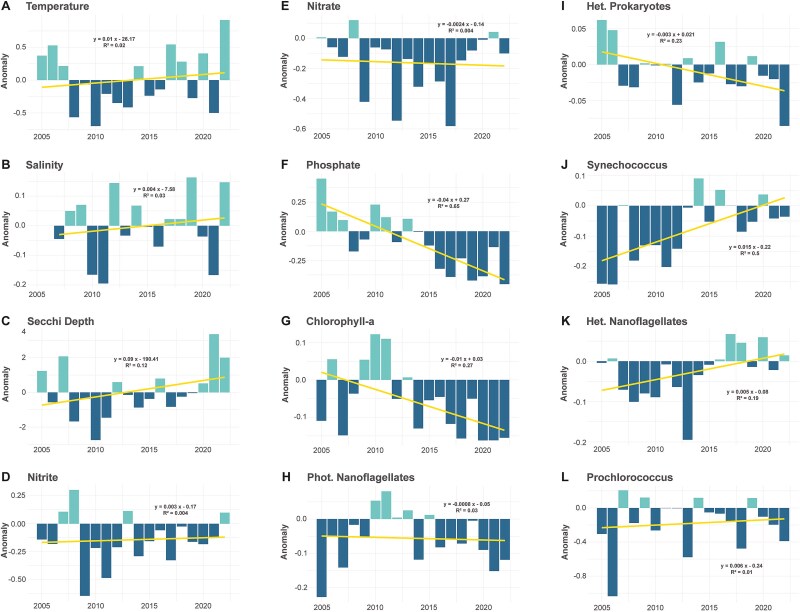
Annual anomalies of various environmental and biological variables in the time series from 2005 to 2022: (A) Temperature, (B) Salinity, (C) Secchi depth, (D) Nitrite, (E) Nitrate, (F) Phosphate, (G) Chl a, (H) PNF, (I) Heterotrophic prokaryotes, (J) *Synechococcus*, (K) HNFs, and (L) *Prochlorococcus*. Bars represent positive and negative anomalies over the average, respectively. The yellow lines in each figure represent the linear regression of the annual anomalies vs. the year.

The Blanes Bay system is characterized by marked seasonality, with consistent temperature variations, ranging from an averaged minima in February of 13.0 ± 0.13°C (mean ± SD) up to averaged maxima in August of 24.71 ± 0.25°C (Supplementary Fig. S4). This cyclic pattern is captured by the GAMM, where the seasonal effect is highly significant (Fig. 3A). Furthermore, GAMMs also revealed an overall warming trend, notably accentuated since 2011, reaching maximal historic temperatures in 2022 with 26.76°C (Supplementary Fig. S4A). This warming tendency is also apparent in the annual anomaly plots (Fig. 4A). Salinity analyses revealed a small yet significant (Supplementary Table S1) seasonal pattern but no interannual changes (Fig. 3B and 4B). Similar to temperature, water transparency (indicated by Secchi Disk depth) showed strong seasonality. Higher values of Secchi depth measurements were observed during summer months, leading to mean annual maxima values of 18.3 ± 0.43 m in September (Fig. 3C), and minimum values of 12.54 ± 0.66 m in February. Alongside this pronounced seasonal variation in water transparency, interannual effects in GAMMs, and anomaly analyses highlighted a consistent, gradual increase in transparency throughout the past two decades (Figs 3C and 4C). In the year of 2021, the highest mean transparency values were observed (18.67 ± 0.35 m).

Regarding inorganic nutrients, both nitrite and phosphate displayed very strong parallel seasonality, peaking in February up to mean values of 0.32 ± 0.03 μmol l^−1^ and 0.12 ± 0.02 μmol l^−1^, respectively (Fig. 3D and F). Nitrite concentrations reached annual average minimum levels in August, dropping to 0.09 ± 0.02 μmol l^−1^, whereas phosphate exhibited lower mean values in July of 0.06 ± 0.01 μmol l^−1^, varying slightly depending on the year. Likewise, the GAMM seasonal component for nitrate revealed a peak in March at 2.62 ± 0.39 μmol l^−1^, followed by a decline during the summer months (July–September) to a minimum of 0.06 ± 0.07 μmol l^−1^ (Fig. 3E). Anomaly analyses and interannual GAMMs revealed a consistent decrease in phosphate concentrations (Figs 3F and 4F). Nitrite concentrations showed a significant decline followed by a rapid increase (Fig. 3D), while nitrate followed a similar, albeit non-significant, pattern (Supplementary Table S1, Fig. 3E). Overall, these results point to a shift toward lower nutrient concentrations, particularly evident in anomaly analyses for the three nutrients considered (NO_3_^−^, NO_2_^−^, and PO_4_^3−^; Fig. 4D–F).

Along the time series, the seasonality of the mean Chl a concentration was similar to that observed for inorganic nutrients, with values ranging from 1.13 ± 0.08 mg m^−3^ in February to 0.28 ± 0.13 mg m^−3^ in August (Fig. 3G). As for the long-term trends, both interannual models and anomaly analysis showed almost constant decay of Chl a concentration during the analyzed period (Figs 3G and 4G). Finally, when looking at the overall seasonal variability, boxplots show a range of variability almost five times higher during the winter months (December–February; SD = 0.48), when chlorophyll generally peaks, than in warmer months (June–August: SD = 0.11), when variability was minimal (Supplementary Fig. S4G).

### Seasonal and long-term temporal trends in microbial abundances

Microbial abundances exhibited a clear seasonal pattern, with different microbial groups reaching their annual peaks in successive months. PNF peak early in the year (1.24 × 10^4^ ± 1.23 cells ml^−1^), coinciding with high inorganic nutrients and Chl a, and drop to a minimum between August and September (2.15 × 10^3^ ± 0.3 cells ml^−1^), depending on the year (Supplementary Fig. S4H). As occurred with Chl a, the variability is larger in the colder months (SD = 4.68 × 10^3^) than in the warmer months (SD = 1.3 × 10^3^). Long-term GAMM components pointed to a decreasing, significant trend in the abundance of PNFs (Fig. 3H) in the last decade, supported by predominantly negative anomalies that indicate this general decline (Fig. 4H).

The seasonal trends of heterotrophic prokaryotes and *Synechococcus* both showed an initial peak in April (Fig. 3I and J), just before the peak in viral abundance in May. In heterotrophic prokaryotes, this April peak (1.02 × 10^6^ ± 0.53 cell ml^−1^) was the most pronounced, followed by a stabilization during summer (9.03 × 10^5^ ± 0.26 cell ml^−1^; July–September) and a decline to a January minimum (7.04 × 10^5^ ± 0.23 cell ml^−1^; Fig. 3I and Supplementary Fig. S3I). In contrast, *Synechococcus* exhibited a different pattern. While it also peaked in April (4.13 × 10^4^ ± 0.76 cell ml^−1^), its most prominent increase occurred in August, when abundances were even higher (4.19 × 10^4^ ± 0.42 cell ml^−1^; Fig. 3J and Supplementary Fig. S3J) and persisted for a longer period. This contrast suggests that heterotrophic prokaryotes exhibit a seasonal pattern more closely aligned with viral abundances, both showing an early spring peak followed by stabilization or decline, unlike *Synechococcus*, which maintains high levels into late summer (Fig. 1). The first yearly peaks of heterotrophic prokaryotes and *Synechococcus* occurred just after the maximum of PNF and winter nutrient concentrations, although *Synechococcus* remained significantly high throughout the year, except during the winter months. Long-term trends showed a significant interannual decline in heterotrophic prokaryotes (Fig. 3I), with a negative anomaly trend (Fig. 4I). Yet, no significant long-term trend was found in *Synechococcus* (Figs 3J and 4J). Temporal models for *Prochlorococcus* show significant seasonal variation, peaking later in the year, reaching abundances of 2.25 × 10^4^ ± 0.17 cells ml^−1^ in September, and dropping in June when they display lowest values (often undetectable, 1.21 × 10^3^ ± 0.27 cell ml^−1^; Supplementary Fig. S4M). GAMM analyses suggested a significant increase in *Prochlorococcus* abundance over the years, with maxima toward the end of the time series (Fig. 3L), which is consistent with the upward tendency that can be observed in anomaly analyses (Fig. 4L).

The seasonal decline in heterotrophic prokaryote and viral abundances was followed by a summer increase in HNF, peaking in June–July (1717 ± 98 cells ml^−1^) before dropping to a January minimum (820 ± 249 cells ml^−1^; Fig. 3K and Supplementary Fig. S3K). Contrary to PNFs, when studying HNF interannual variability, a significant positive trend was observed since the end of 2011, which also aligned with the anomaly observations (Fig. 4K).

### Modeling the response of viral abundances to environmental and biotic variables

**Figure 5:**
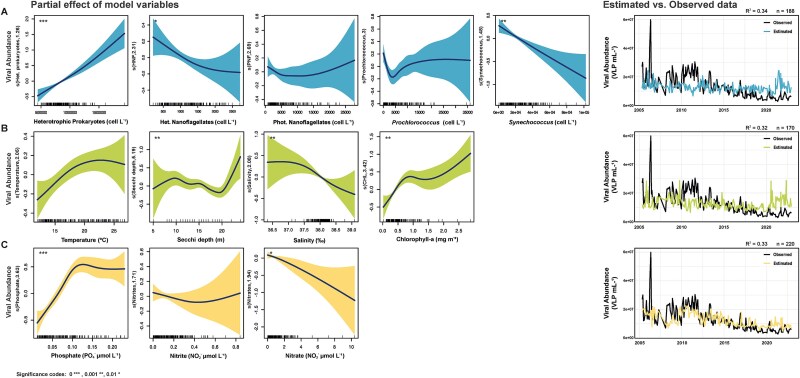
Partial effects of three predictor variable groups on viral abundances (left panels): (A) putative host abundances (blue), (B) water mass characteristics (green), and (C) nutrients (yellow). The GAMMs plots show smoothed partial effects alongside observed viral abundance values (VLP ml^−1^), with shaded areas representing 95% confidence intervals for the smoothing splines. The significance of each term is denoted using *P*-value codes (^***^P < .001, ^**^P < .01, ^*^P < .05, *P* < .1). On the right, the plots display the observed vs. model estimated viral abundances values for each group of variables across the dataset from 2005 to 2022.

Viruses likely interact with both their environment and the other microorganisms [40], however, partial effects of each variable are often intertwined due to high correlations between them. This complexity is also evident in Supplementary Fig. S5, where nutrient-related variables and temperature appear not only distinct (Supplementary Fig. S5A) but also negatively intercorrelated (Supplementary Fig. S5B), suggesting that viruses may respond to a network of co-varying environmental and biological factors. For instance, phosphate and chlorophyll-a were strongly positively correlated, while temperature showed negative correlations with both. In order to disentangle these effects, we generated GAMMs based on the partial effect of three distinct blocks of variables on viral abundance: (i) potential host abundances, (ii) water mass characteristics, and (iii) inorganic nutrients (Fig. 5).

Regarding the virus-host GAMM, heterotrophic prokaryotes, and *Synechococcus* abundances emerged as key predictors of viral abundances (Fig. 5A). While the association between viruses and heterotrophic bacteria was positive, the association with *Synechococcus* was negative. Additionally, the partial effect of HNF was significant, revealing a slightly negative association between viral and HNF abundances, which could derive from the negative association between HNFs and heterotrophic prokaryotes. The second model explored the influence of water mass characteristics on viral abundances (Fig. 5B). Among these, temperature and Chl a concentration showed a positive association with viral abundances, although only the later association was statistically significant (Supplementary Table S2). Meanwhile, the associations of viral abundance with salinity and water transparency were significant, but no clear pattern could be established in the trends. Finally, the last GAMM highlighted the role of nutrients in driving viral abundances (Fig. 5C). On the one hand, phosphate concentration had a significant positive association with viral abundance. However, at high phosphate levels, this effect plateaued, indicating a saturation point beyond which viral abundance no longer increased. On the other hand, nitrate exhibited a negative association with viral abundances, whereas nitrite was relatively neutral. The three partial models explained ~32%–34% of the variance (Supplementary Table S2; Fig. 5A–C).

**Figure 6:**
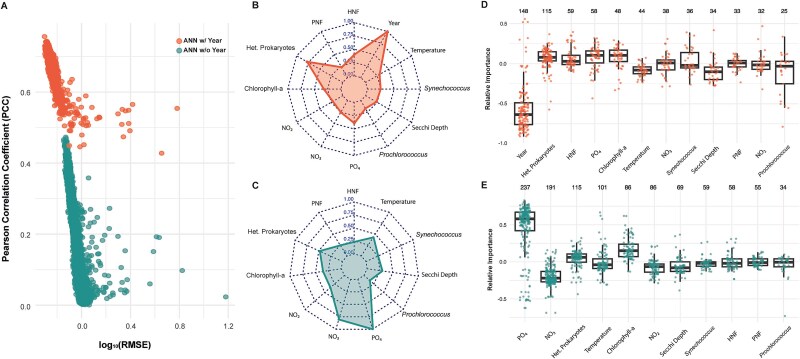
Best predictor variables in ANNs shaping viral abundances. (A) Dispersion plot showing the log_10_ RMSE vs, PCC for all ANN models across 7722 different predictor combinations. The top (orange) point cluster represent models that include the long-term temporal predictor (Year), while the bottom (green) point cluster represent models without it. (B) Frequency of inclusion of each variable among the top 5% best-performing models (i.e. PCC > 0.7) that include Year as a predictor. (C) Frequency of inclusion of each variable among the top 5% best-performing models (i.e. PCC < 0.5) that exclude year. (D) Boxplots showing the relative importance of each predictor in the top 5% of models that include Year. (E) Boxplots showing the relative importance of each predictor in the top 5% of models that exclude Year. In boxplots, the top number represents the number of times that predictor appears in a model.

We also evaluated the use of ANNs to describe the temporal dynamics of viral abundance using the environmental and biotic variables. The aim here was to identify those variables that had the strongest relevance shaping viral abundance (Fig. 6). Among the 7722 tested neural networks (Supplementary Table S3; Fig. 6), the network that provided the most accurate predictions of viral abundance achieved a high Pearson Correlation Coefficient (PCC = 0.76) and a low Root Mean Square Error (RMSE = 0.5). Using the Olden’s method we estimated each predictor importance in the ANN, assigning values based on their influence on the model’s output. Positive values indicate positive effects on viral abundance, and vice-versa, with larger magnitudes indicating greater importance. The best ANN identified Year as the strongest predictor with a dominant negative effect (−0.8), capturing the decline in viral abundance (Supplementary Table S3). Among positive predictors, nitrate (0.07) had the highest impact, followed by heterotrophic prokaryote (0.06) and HNF abundance (0.06).

When the temporal factor (Year) is assessed in the 7722 neural networks (Fig. 6A) the performance, here intended as PCC, improved by more than 20%. Considering all the analyzed ANNs containing Year as a predictor, we selected the best 5% based on the coefficient of determination (PCC) and then explored their potentially relevant variables. Since ANNs included multiple predictors, we calculated the normalized frequency of each predictor in the top 5% selected models (Fig. 6B). Heterotrophic prokaryote abundance was the second most often predictor included in the top-scoring ANNs (78%), followed by HNF abundance (40%), phosphate concentration (40%), Chl a concentration (32%), and temperature (30%; Fig. 6B). Conversely, when the temporal component (*Year*) was excluded, the performance of the ANNs dropped significantly, with the maximum PCC reaching only 0.48 (Fig. 6A). Under these conditions, phosphate and nitrate concentration emerged as the most frequent predictors. Phosphate concentration was included in all of these models, while nitrate concentration appeared in 80.5% of them, followed by heterotrophic bacterial abundance (48.5%), temperature (42.6%), Chl a (36.3%)*,* and nitrite concentration (36.3%; Fig. 6C).

The relative importance of predictors in the top 5% of neural networks highlights their roles in shaping viral abundance (Fig. 6D). The temporal factor (*Year*) showed a predominantly negative effect (median importance: −0.64), while heterotrophic bacterial abundance consistently had a positive influence. Without the temporal component, phosphate concentration emerged as the strongest positive predictor (median importance: 0.58), followed by nitrate concentration with a negative effect (Fig. 6E). Similarly, bacteria maintained a positive impact in the models explaining viral abundance, while temperature mostly showed a negative influence.

## Discussion

This study identifies clear seasonal patterns in the Blanes Bay system, marked by significant variations in surface seawater parameters. Our observations confirm seasonal dynamics inherent to similar coastal systems [13, 41], with a clear succession of different microorganisms throughout the year and a pronounced seasonality, consistent with findings from other studies in the area [42–44]. Initial peaks in heterotrophic prokaryotes in April were followed by increases in viral abundance with bacteria likely serving as the primary hosts for viruses [14, 15, 17, 45]. Although this sequential order may suggest that viruses regulate host populations, in oligotrophic systems like Blanes Bay, it is host abundance and metabolic activity—constrained by nutrient availability—that primarily govern viral dynamics. GAMMs further reveal possible interactions between viruses and *Synechococcus*, as the second peak in viral abundance typically follows the summer rise in *Synechococcus* abundance, which reaches its peak just before the viral increase. This suggests a possible link, potentially driven by specific viruses infecting this cyanobacterial population, as previously reported in Marston et al. (2003) [46]. On the other hand, negative relationship between viruses and HNF observed in the GAMMs could stem from multiple factors. One possibility is competition for bacterial prey, as both groups rely on prokaryotes—viruses through infection and HNFs through predation. This competition is further exacerbated by the progressive decrease in prokaryotes over time, which we have highlighted and which represents a resource limitation for both [47]. Another possible explanation derives from the fact that HNF abundance has increased while viral abundance has declined during the last decade. This was further corroborated by neural networks, where in the top 5% of models that include *Year* as a predictor, HNF ranks as the third most frequent predictor with positive relative importance. However, when excluding *Year*, HNF falls to the ninth position, appearing in only a small proportion of the models. This suggests that long-term temporal trends could be influencing how GAMMs interpret the viral-HNF relationships, unlike neural networks, which do not account for this and therefore may provide a better fit.

The well-defined seasonal succession in microbial communities in Blanes Bay is typical of oligotrophic environments, where both abundance and activity of microorganisms increase with competition for a shared limiting resource (mostly phosphate in our case). This was demonstrated by Guadayol et al. (2009) [48], who observed the direct relationship between nutrient availability and variations in phytoplankton abundance. Even though nutrient concentrations are generally low in Blanes Bay, particularly in summer [44, 48, 49], the observed continuous decrease in nitrite and phosphate together with the decrease in Chl a concentration suggests an ongoing oligotrophication process. This has been previously reported not only in Blanes [42] but throughout the Mediterranean [50–52]. In fact, Agusti et al. (2017) [41] reported a significant ongoing decay in both nutrient and consequent Chl a concentration, representing a loss of 4.5% of Chl a per year between 2006 and 2015 in a coastal site in Mallorca, Balearic Islands. In our research, we also observed a gradual increase in water transparency alongside the decline in Chl a concentration, which suggests a decrease in overall productivity, possibly due to a reduction in phytoplankton stock/biomass. This decline in productivity and nutrient availability, particularly phosphorus, can significantly influence various aspects of viral ecology, such as viral abundance [14, 45], life cycles [53, 54] and burst size [55, 56]. Blanes Bay has experienced a notorious decrease in viral abundance starting in 2012, reaching its lowest levels by the end of the time series. In fact, this study also identified a tipping point in 2011–2012, from which the seasonality of viral abundance was attenuated until the end of the time series. Both neural network analyses and temporal GAMMs highlighted this long-term effect as a significant factor in this time series. Interestingly, our models also suggest a gradual decline in inorganic nutrient levels, particularly phosphorus. Since viral replication and life cycles are connected to host metabolism [40], changes in nutrient availability would affect hosts and thus, viruses. In fact, Wilson et al. (1996) [53] observed that phosphate limitation reduced viral lysis by 90%, favoring lysogeny compared to phosphate-enriched conditions. Moreover, nutrient limitation could have significant consequences not only on host physiology but also on the burst size of infected cells [56]. Maat & Brussaard (2016) [55] found that nitrogen and phosphorus limitation prolong viral latency periods and dramatically reduces viral burst size. In other words, fewer viral particles are produced, leading to fewer future infections. When the ecosystem shifts toward lower bacterial density and higher oligotrophication, viruses tend to enter latency phases, increasing levels of viral lysogeny [9]. A previous study on nutrient stoichiometry and viruses revealed that phosphorus scarcity results in lower viral production, as viruses can use up to 87% of a host cell’s intracellular phosphorus during replication [57]. In our study, while causality cannot be inferred, models reveal strong co-variation patterns that suggest nutrient availability plays a key role in shaping viral dynamics. However, this high demand for phosphorus by the virus-host system, could make viruses particularly vulnerable to global change scenarios projected for the Mediterranean region [5], particularly in the North-Western Mediterranean where phosphorus decrease is reported to be more acute [52].

In our data, temperature remains one of the most important predictors of viral abundance in neural network models, even when the temporal component is removed. This, together with the observed increase in temperature and concurrent decrease in viral abundance over the last decade, supports the hypothesis that temperature may exert both direct and indirect influences on viral dynamics. Directly, temperature affects viral processes such as infection, replication, and burst size [58], and also influences host metabolism [59], which can impact virus production. Indirectly, as viruses are obligate parasites, their success depends on the physiological state of their hosts and broader environmental conditions [60]. This complexity is illustrated by Vaqué et al. (2019) [61], who observed that temperature increases in the Arctic Ocean may enhance bacterial production—likely triggered by shifts in primary producers—and promote lysogeny over viral lysis. Therefore, the overall effect of temperature on viruses may be more intricate than expected. In fact, Wieczynski et al. (2023) [58] compiled evidence of opposing effects of temperature at different stages of the viral life cycle, affecting infection efficiency, replication rates, and the likelihood of lysogeny. Different ocean regions are expected to respond differently to rising surface water temperatures, particularly temperate warm oceans, likely leading to variations in viral abundance [62]. Furthermore, a recent article on seasonal data from eight prokaryotic time series in both the northern and southern hemispheres [63] suggests that factors such as daylength, rather than temperature alone, may play a key role in shaping the seasonal diversity and ecological rhythms of prokaryotic microbes, and, by extension, of viruses.

Similar to viral particle abundances, viral community composition also displayed significant seasonal variation, with notable differences between summer and winter communities, likely driven by temperature fluctuations. Other studies in the area observed that bacterial communities [64] and even phylogroups [65] exhibited distinct seasonal behaviors, with *Synechococcus* more abundant during warmer months, and SAR11 dominating colder months. This, in association with the oligotrophic conditions observed among summer months, suggests the shifts in viral community composition observed at this time.

## Conclusions

Our findings highlight the complex interplay between environmental factors, microbial dynamics, and viral communities in Blanes Bay, revealing long-term ecological shifts driven by climate change. We provided evidence of the seasonal dynamics of microbial and viral communities in Blanes Bay, highlighting long-term environmental changes shaping these. The impact of global change, such as surface waters warming and nutrient decrease, is likely to intensify its effects on host-virus dynamics. Specifically, the observed decrease in viral abundance, along with a potential increase in lysogeny under oligotrophic conditions, suggests a shift in viral life strategies. In nutrient-poor environments, as reported in the literature, viruses may more frequently adopt lysogenic cycles, thus reducing their immediate impact on microbial mortality. This shift could alter the balance between lytic and lysogenic interactions, leading to more stable but less dynamic microbial communities. As environmental pressures such as warming and nutrient depletion intensify, viruses may transition from active agents of microbial turnover to more passive genetic reservoirs, fundamentally changing their role in microbial population control and biogeochemical cycling. Understanding these changes is crucial to ensure the future stability of coastal ecosystems in face of global change.

## Supplementary Material

Supplemental_Information_def_26_08_2025_ycaf150

## Data Availability

All code is available at https://github.com/xabilopalf/GAMMANN-Vir.git
