# Supplementary material for: Long-term decline of marine viruses associated with warming and oligotrophication at a NW Mediterranean coastal site
Source: ISME Commun. 2025 Aug 29;5(1):ycaf150. doi: 10.1093/ismeco/ycaf150 (PMC12452277; doi:10.1093/ismeco/ycaf150)
Supplement: Supplemental_Information_def_26_08_2025_ycaf150 [file supplemental_information_def_26_08_2025_ycaf150.docx]

Supplemental Information for:

**Long-term decline of marine viruses associated with warming and oligotrophication at a NW Mediterranean coastal site**

Xabier Lopez-Alforja^(1,2)*^, Elisabet L. Sà^(1)^, Maria V. Quiroga ^(3)^, Massimo C. Pernice^(1)^, Clara Cardelús^(1)^, Vanessa Balagué^(1)^, Josep M. Gasol^(1)^, Felipe H. Coutinho^(1)^, Ramon Massana^(1)^ & Dolors Vaqué^(1)^

^(1)^ Department of Marine Biology and Oceanography, Institut de Ciències del Mar (ICM-CSIC), 08003, Barcelona, Catalonia, Spain

^(2)^ Department of Genetics and Microbiology, Autonomous University of Barcelona, 08193 Bellaterra, Catalonia, Spain

^(3)^ Instituto Tecnológico de Chascomús (CONICET-UNSAM), Escuela de Bio y Nanotecnologias (UNSAM), 7130, Chascomús, Buenos Aires, Argentina

**Corresponding author* : Xabier López-Alforja *(*[*xabierlopez@icm.csic.es*](mailto:xabierlopez@icm.csic.es)*)*

**Description of Supporting information content:**

All supporting information is compiled into a single file. including the following. in order:

- 3 supplementary tables (2 inside this document. and 1 attached)
- 5 supplementary figures
- Protocol for RAPD-PCR for dsDNA Viral Diversity Analysis
- Artificial Neural Networks (ANN) for Viral Abundance Modelling

**Table of Contents:**

| **Tables:** | | |  | |  |
| --- | --- | --- | --- | --- | --- |
|  | Table S1 | ……………………………….……………………………….………………  ……………………………….……………………………….……………… | | | Page 3 |
|  | Table S2 | ……………………………….……………………………….……………… | | | Page 4 |
|  | Table S3 | ……………………………….……………………………….……………… | | | *Attached* |
|  | | |  | |  |
| **Figures:** | | |  | |  |
|  | Figure S1 | | ……………………………….……………………………….………..………  ……………………………….……………………………….……………… | | Page 5 |
|  | Figure S2 | | ……………………………….……………………………….………..……… | | Page 6 |
|  | Figure S3 | | ……………………………….……………………………….………..……… | | Page 7 |
|  | Figure S4 | | ……………………………….……………………………….………..……… | | Page 8 |
|  | Figure S5 | | ……………………………….……………………………….………..……… | | Page 9 |
|  |  | | | |  |
| **Protocols:** | | |  | |  |
| RAPD-PCR protocol | | | | ……………………………….……………………………….……………… | Page 10 |
| ANN Analysis | | | ……………………………….……………………………….……………… | | Page 12 |

|  | |  | |  |  | | |  | | |  | |  | | **Temporal Partial Models** | | | |  |
| --- | --- | --- | --- | --- | --- | --- | --- | --- | --- | --- | --- | --- | --- | --- | --- | --- | --- | --- | --- |
|  | | | **n** | **mean** | **median** | **sd** | **min** | | **max** | **range** | | **se** | | **s(Month)** | | **s(Trend)** | **ti()** | **Adj-R^2^** | |
| Temperature (ºC) | | | 258 | 17.91 | 17.11 | 4.30 | 11.94 | | 26.76 | 14.82 | | 0.27 | | <2e-16 *** | | 0.038 * | - | *0.93* | |
| Salinity (‰) | | | 230 | 38.00 | 38.06 | 0.29 | 36.29 | | 39.09 | 2.80 | | 0.02 | | 0.00154 ** | | - | - | *0.07* | |
| Secchi depth (m) | | | 234 | 15.46 | 16.00 | 3.66 | 5.00 | | 24.00 | 19.00 | | 0.24 | | <2e-16 *** | | 0.003 ** | - | *0.22* | |
| [NO₃⁻] (μmol L⁻¹) | | | 258 | 0.99 | 0.53 | 1.30 | 0.00 | | 10.39 | 10.39 | | 0.08 | | <2e-16 *** | | - | - | *0.20* | |
| [PO₄⁻] (μmol L⁻¹) | | | 258 | 0.08 | 0.06 | 0.06 | 0.01 | | 0.47 | 0.46 | | 0.00 | | <2e-16 *** | | <2e-16 *** | - | *0.60* | |
| [NO₂⁻] (μmol L⁻¹) | | | 258 | 0.16 | 0.12 | 0.15 | 0.00 | | 0.99 | 0.99 | | 0.01 | | <2e-16 *** | | 0.098 . | - | *0.25* | |
| Chlorophyll a (mg m⁻³) | | | 253 | 0.54 | 0.39 | 0.44 | 0.02 | | 2.88 | 2.86 | | 0.03 | | <2e-16 *** | | 0.0007 *** | 0.029 * | *0.40* | |
| Het. Bacteria (cell ml⁻¹) | | | 229 | 8.65·10^5^ | 8.39·10^5^ | 2.23·10^5^ | 4.13·10^5^ | | 1.94·10^6^ | 1.53·10^6^ | | 1.47·10^4^ | | 3.51e-06 *** | | 0.009 *** | 0.003 * | *0.19* | |
| *Prochlorococcus* (cell ml⁻¹) | | | 241 | 7.76·10^3^ | 4.11·10^3^ | 1.33·10^4^ | 0.00 | | 1.28·10^5^ | 1.28·10^5^ | | 8.54·10^2^ | | <2e-16 *** | | 0.00121 ** | - | *0.30* | |
| *Synechococcus* (cell ml⁻¹) | | | 244 | 2.50·10^4^ | 2.14·10^4^ | 1.87·10^4^ | 6.72·10^2^ | | 1.33·10^5^ | 1.32·10^5^ | | 1.20·10^3^ | | <2e-16 *** | | - | - | *0.36* | |
| Het. Nanoflagellates (cell ml⁻¹) | | | 228 | 1.18·10^3^ | 1.15·10^3^ | 5.92·10^2^ | 2.10·10^2^ | | 4.53·10^3^ | 4.32·10^3^ | | 39.2 | | <2e-16 *** | | 0.0274 * | - | *0.41* | |
| Phot. Nanoflagellates (cell ml⁻¹) | | | 232 | 5.31·10^3^ | 3.38·10^3^ | 4.76·10^3^ | 7.76·10^2^ | | 2.78·10^4^ | 2.70·10^4^ | | 3.13·10^2^ | | <2e-16 *** | | 0.009 *** | - | *0.54* | |
| Virus Total (VLP ml⁻¹) | | | 220 | 1.23·10^7^ | 1.06·10^7^ | 7.78·10^6^ | 2.77·10^6^ | | 5.99·10^7^ | 5.71·10^7^ | | 5.24·10^5^ | | 0.00279 ** | | <2e-16 *** | 0.0094 ** | *0.65* | |
|  | |  |  |  |  |  | |  |  | |  | |  | |  |  |  | |  |

**Table S1.** Summary of descriptive statistics and results of the temporal partial models for environmental and biological variables. Number of observations (n); mean; standard deviation (sd); minimum (min); maximum (max); range; standard error (se) and time range for each variable. The temporal partial Generalized Additive Mixed Model (GAMM) examine the smooth functions of month (s(Month)), Long-term trend (s(Trend)), and the interaction between Month and Long-term Trend (ti()), and the adjusted R-squared (Adj-R^2^). Significance codes: *** *p* < 0.001, ** *p* < 0.01, * *p* < 0.05, **^·^** *p* < 0.1.

| **Virus Abundance** | **A)** | **s(Het_Prok)** | **s (HNF)** | **s(PNF)** | **s(*Prochlorococcus)*** | **s(*Synechococcus*)** | Adj-R^2^ | n |
| --- | --- | --- | --- | --- | --- | --- | --- | --- |
|  |  | 1.25·10^5^ *** | 0.0371 * | - | 0.0258 * | 8.72·10^5^ *** | 0.334 | 188 |
|  |  |  |  |  |  |  |  |  |
|  | **B)** | **s(Salinity)** | **s(Secchi Depth)** | **s(Temperature)** | **s(CHL)** |  |  |  |
|  |  | 0.00702 ** | 0.00429 ** | - | 0.00116 ** |  | 0.319 | 170 |
|  |  |  |  |  |  |  |  |  |
|  | **C)** | **s(PO_4_)** | **s(NO_2_)** | **s(NO_3_)** |  |  |  |  |
|  |  | <2·10^16^ *** | - | 0.0103 * |  |  | 0.329 | 220 |

**Table S2.** Summary of three partial Generalized Additive Mixed Models (GAMM) with viral abundance as a response variable A) putative host abundances. B) water mass characteristic variables, and (C) nutrients. Significance of the smooth functions for each variable are shown, as well as the adjusted R-squared (Adj-R^2^). Significance codes: *** *p* < 0.001. ** *p* < 0.01. * *p* < 0.05. **^·^** *p* < 0.1.

**
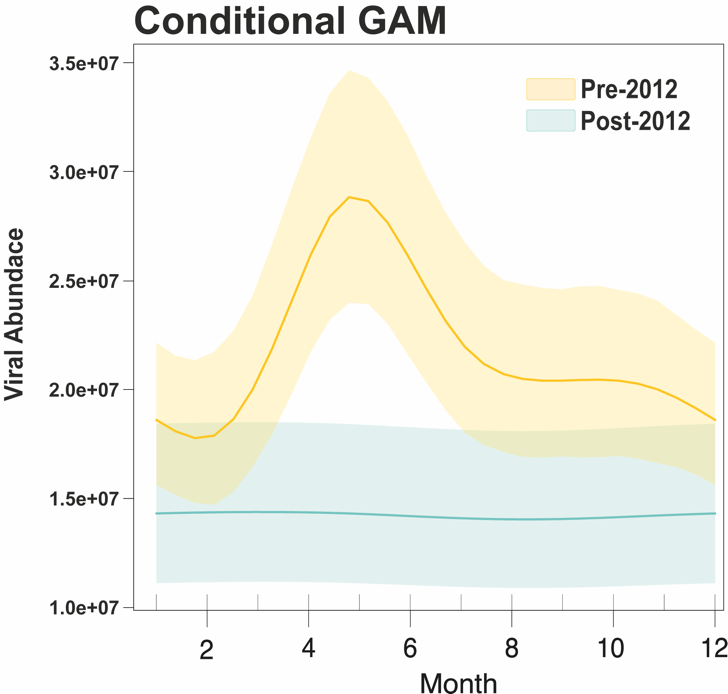
**

**Figure S1.** GAMM analyzing viral population fluctuations influenced by seasonality and climate dynamics. The model included the 2011-2012 inflection point as a constraint on the seasonality smoothing parameter, but this did not improve the fit (R² = 0.63). Before 2012 seasonality was significant (⍺ < 0.001), but after 2012 became non-significant (⍺ = 0.47). Model performance improved with the Akaike Information Criterion (AIC) decreasing from 177 to 167. Partial effects highlighted this shift, incorporating a two-level factor for pre- and post-2012 periods in the month-smooth term. The y-axis shows predicted viral abundance, with shaded regions indicating 95% confidence intervals.

**Figure S2.** Similarity dendrograms calculated by the UPGMA method with software GelJ from banding patterns obtained by RAPD in viral samples collected between 2011 and 2021. For better visualization each panel represents clusters of samples collected in consecutive years: A) 2010-2012. B) 2013. C) 2014. D) 2015-2016. E) 2017. F) 2018-2019. G) 2020-2021. For final comparison we aligned all years together. Ladders indicate molecular weight in base pairs (bp), including 2000 bp, 1000 bp, 500 bp 250 bp, and 100 bp.

**
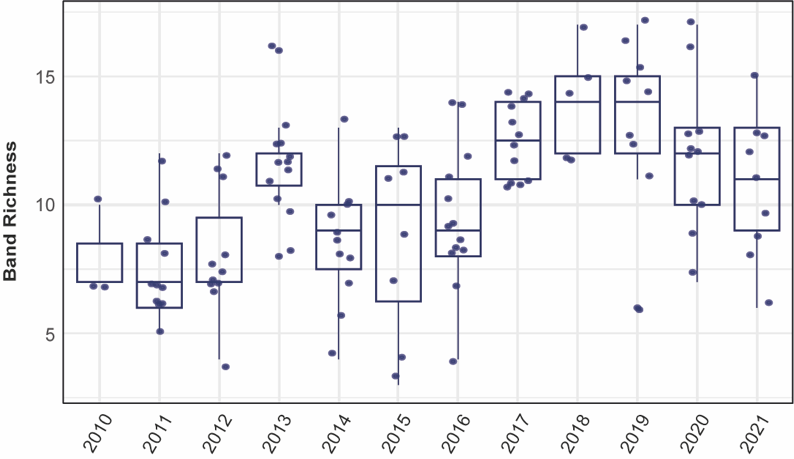
**

**Figure S3.** Boxplot of the band richness of the Random Amplified Polymorphic DNA (RAPD) analysis of 120 samples collected between 2011 and 2021.


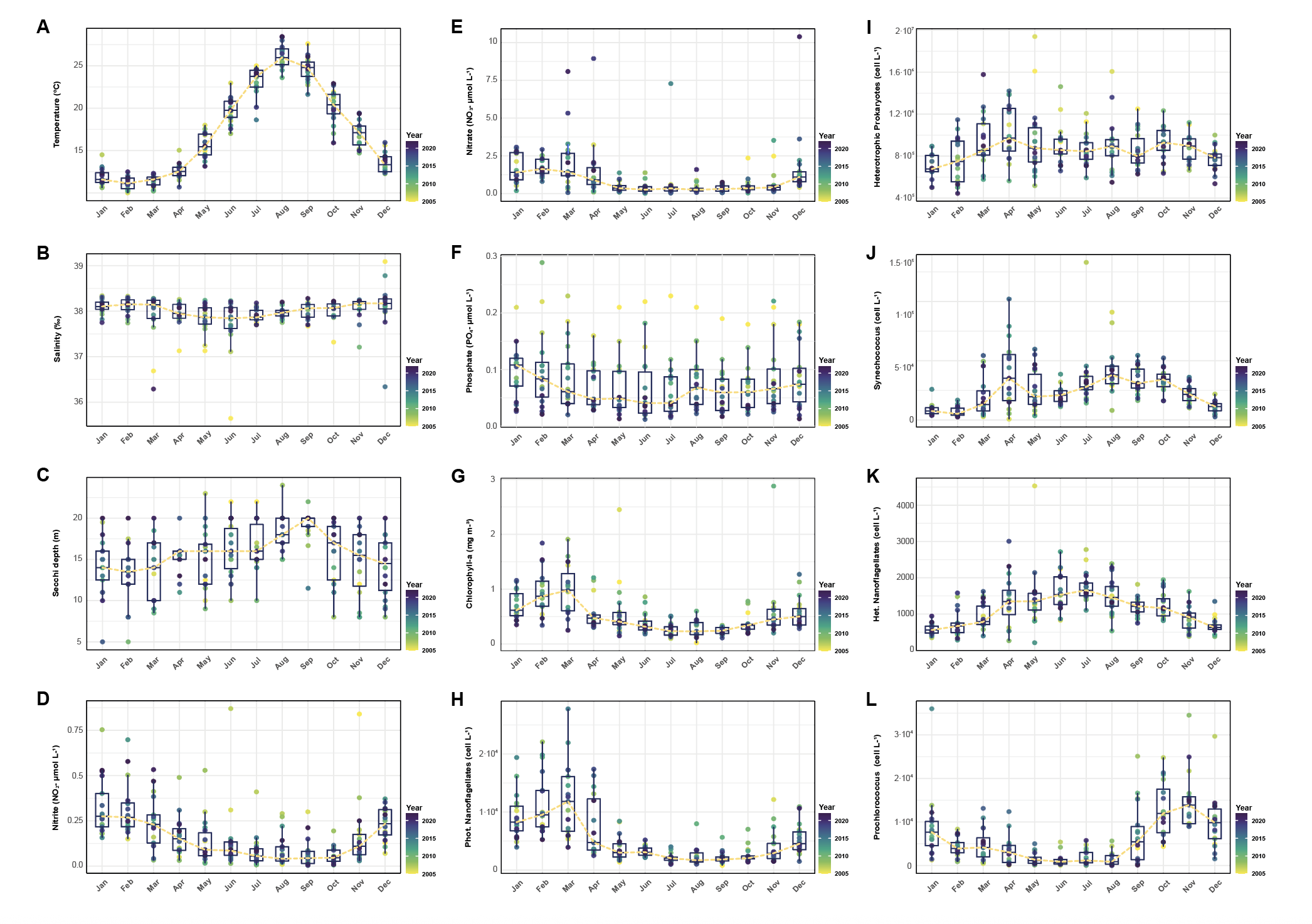


**Figure S4.** Boxplots depicting observed values for different variables categorized by month and colored by year to illustrate the temporal variations over time: A) Temperature, B) Salinity, C) Secchi Depth, D) Nitrite, E) Nitrate, F) Phosphate, G) Chlorophyll *a* concentration, H) Phototrophic Nanoflagellates, I) Heterotrophic prokaryotes, J) *Synechococcu*s, K) Heterotrophic Nanoflagellates, and L) *Prochlorococcus* abundances. Each boxplot summarizes the distribution of the time series values for a specific month. with the central line indicating the median, the box representing the interquartile range (IQR spanning Q1 to Q3), and the whiskers extending up to 1.5 times the IQR or the most extreme data points within that range. Data points falling outside this range are displayed as outliers.


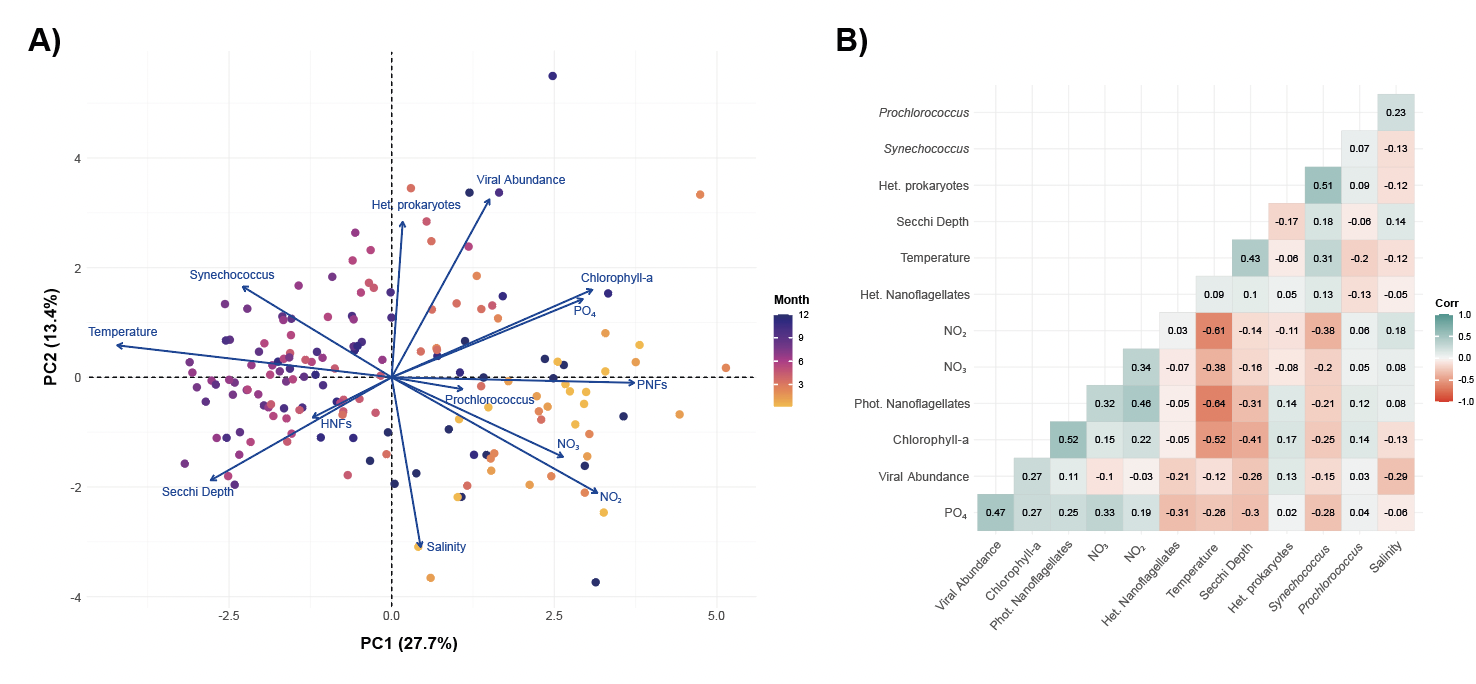


**Figure S5.** A) Principal Component Analysis (PCA) plot of the distribution of the samples and variables studied. The plot shows the distribution of the variables in the first two principal components. Axes represent the percentage of variance explained by each component: the first component (PC1) explains 27.7% of the variance, while the second component (PC2) explains 13.4%. Color of the points represents the Month of the samples, and the arrow size of the arrows represents the contribution of the variables to the principal components. B) Correlogram of the variables. The plot shows the correlation between the variables in the dataset. Type of correlation test used in the correlogram is Pearson correlation. Square colors represent strength and direction of the correlation, as size of the squares represents the magnitude of the correlation. Variables are ordered by hierarchical clustering.

**Supplementary Protocol: RAPD-PCR for dsDNA Viral Diversity Analysis**

**Sample Collection and Viral Concentration**

From September 2010 to December 2021, a total of 120 seawater samples were analyzed using randomly amplified polymorphic DNA-polymerase chain reaction (RAPD-PCR) as a method for measuring dsDNA viral diversity. Here, we use RAPD profiles as a proxy to assess temporal changes in viral community composition, acknowledging that the observed patterns are shaped by the technique’s inherent biases—particularly its preferential amplification of dsDNA viruses—and should be interpreted within these methodological constraints.

Each month, 5L of seawater were collected and sequentially filtered through 3.0 µm and 0.22 µm filters. Viruses were then concentrated from the 0.22 µm filtrate by using tangential flow filtration (30 KDa VIVAFLOW 200) until reaching a final volume of 50 ml.

This viral concentrate was further filtered through a 0.2 µm polycarbonate nucleopore filter to remove any remaining prokaryotic cells that could contribute non-viral DNA to the banding pattern. The final concentrate was ultra-concentrated to 400 µL by centrifugation at 4500 rpm for 10 minutes in 30-kDa ultracentrifuge filter tubes (Amicon. Sigma), following the protocol described by Winget & Wommack (2008) [1].

To prevent changes in viral community composition, the concentrated viral samples were stored in agar plugs. Equal volumes of viral concentrate and melted 1.6% low‐melting‐point agarose (Pronadisa) were mixed, transferred to plug molds, and allowed to solidify at room temperature. The solidified plugs were then stored in TE (Tris-EDTA) buffer at 4°C until further analysis.

**Prokaryotic DNA Removal and Viral DNA Extraction**

Prior to DNA amplification, the absence of DNA of the prokaryotic origin was verified by PCR of the 16S rRNA gene to ensure that samples were free of bacterial contamination. If contamination was detected, the sample was treated with DNAse and rechecked.

Viral DNA was extracted from one agar plug per sample by overnight incubation at 50°C in ESP buffer (0.5M EDTA. pH 9.0. 1% N‐laurylsarcosine. and 1 mg mL⁻¹ proteinase K (Sigma)) to break down viral capsids. The sample was then treated with 3mM Pefabloc® (Sigma) to inactivate proteinase K, followed by several washes with TE and low-EDTA TE to remove any remaining Pefabloc.

**RAPD-PCR Amplification**

Molten and contamination-checked agar plugs were used as templates for RAPD-PCR. The decamer CRA22 (5’-GCG ATC CCC A-3’) was used as both forward and reverse primer. PCR conditions were:

- 1 cycle of 10 min at 94°C
- 30 cycles of:
  - 3 min at 35°C
  - 1 min at 72°C
  - 30 s at 94°C
- 1 cycle of 3 min at 35°C
- 1 cycle of 10 min at 72°C (final extension)

**Electrophoresis and Gel Imaging**

RAPD-PCR products were stained with 5 µL of SYBR Safe (10.000X concentrate in DMSO, Invitrogen) and separated by electrophoresis on 1.8% low-melting-point agarose (Pronadisa) gels prepared in TAE buffer. The gels were run at 70V for 120 minutes and a DNA ladder (100-2000 bp) was loaded in the first and last lanes of each gel.

After electrophoresis, the gel images were captured using the ChemiDoc™ imaging system (Bio-Rad) with an exposure time of approximately 2.1 seconds.

**Data Analysis**

Gel band patterns obtained by RAPD-PCR were analyzed using the open-source software GelJ [2]. The banding patterns were adjusted with the DNA ladders, allowing direct comparison between different gels. Additionally, replicates of selected samples were run across multiple gels to ensure proper alignment and normalization of banding patterns based on the duplicates and the reference ladder.

**Supplementary Protocol: Artificial Neural Networks (ANN) for Viral Abundance Modelling**

**Artificial Neural Network training and optimization**

To model viral abundances using Artificial Neural Networks (ANN), we employed the *nnet* package (version 7.3 [3]) and the *caret* package (version 6.0 [4]). The *nnet* function was used to train neural networks, incorporating physical (e.g., temperature), chemical (e.g., nitrate concentrations), and biological (e.g., heterotrophic nanoflagellates -HNF- and heterotrophic bacterial abundances) parameters as predictor variables.

To implement this approach, we preprocessed the dataset, including a log-transformation of viral and bacterial/prokaryotic abundances. The dataset was then **z-transformed**, standardizing each variable by subtracting the mean and dividing by the standard deviation to improve model convergence.

A **parallelized process** was implemented to evaluate different predictor combinations efficiently, systematically testing models with varying hyperparameters using a **grid search approach**. In this context, hyperparameters refer to model settings defined prior to training—such as the number of training iterations, regularization strength (decay), and the number of neurons in the hidden layer—which influence how the neural network learns and generalizes from the data. Hyperparameter tuning was performed using functions from the caret package, specifically utilizing Leave-Group-Out Cross-Validation (LGOCV) to optimize model performance. This approach ensured a robust selection of model parameters by systematically evaluating different network configurations while avoiding overfitting.

The modeling process was conducted in two main steps. First, multiple ANN models were trained and tested using all possible combinations of three to four predictor variables (e.g., salinity, temperature, chlorophyll *a*, and nutrient concentrations). Each model's performance was assessed using two key metrics: the Pearson correlation coefficient (PCC) and the root mean square error (RMSE), which measure the agreement between observed and predicted viral abundances. Moreover, the relative importance of each predictor variable was calculated using the Olden method, which determines the contribution (Relative Importance) of each predictor to the final ANN model.

Each model evaluation was carried out on two separate subsets of the dataset: a training subset (80% of the data) used to develop the model, and a hold-out test subset (20% of the data) used to assess its generalization capability. An optimal model was characterized by high PCC values (indicating strong correlation with observed data) and low RMSE values (indicating minimal prediction error). Moreover, the **relative importance of each predictor variable** was calculated using the **Olden method**, which determines the contribution (the relative importance) of each predictor to the final ANN model.

**ANN Predictor Frequency & Relative Importance**

Additional analyses were conducted to visualize and assess the influence of predictors on viral abundance estimation. Given the observed differences between models with and without the variable "Year", the frequency of predictor usage was analyzed. The top 5% of models, based on PCC, were selected in both categories to analyze the most frequently used predictors in our ANNs. This approach helped identifying the key environmental and biological variables contributing to viral abundance predictions. Following this idea, the relative importance of each predictor in the best-performing models were evaluated using the Olden’s method. This analysis provided valuable insights into the variability in predictor contributions, highlighting those with the greatest impact on model estimations. Together, these calculations offered a deeper understanding of the factors influencing predictions, aiding in model interpretation and potential refinements.

The main objective of this modeling approach was to develop a final ANN model that not only demonstrated strong predictive performance on the training data but also generalized effectively to new, unseen data. By following this exhaustive selection and validation process, we ensured that the final model provided an accurate and reliable estimation of viral abundances based on the chosen environmental and biological predictor variables.

**References**

1. Winget DM, Wommack KE. Randomly Amplified Polymorphic DNA PCR as a Tool for Assessment of Marine Viral Richness. *Appl Environ Microbiol* 2008;**74**:2612–2618. https://doi.org/10.1128/AEM.02829-07

2. Heras J et al. GelJ – a tool for analyzing DNA fingerprint gel images. *BMC Bioinformatics* 2015;**16**:270. https://doi.org/10.1186/s12859-015-0703-0

3. Ripley B. nnet: Feed-Forward Neural Networks and Multinomial Log-Linear Models. 2009. 2009. , 7.3-20

4. Kuhn M. caret: Classification and Regression Training. 2007. 2007. , 7.0–1
